# Frequent misdirected courtship in a natural community of colorful *Habronattus* jumping spiders

**DOI:** 10.1371/journal.pone.0173156

**Published:** 2017-04-05

**Authors:** Lisa A. Taylor, Erin C. Powell, Kevin J. McGraw

**Affiliations:** 1 Entomology and Nematology Department, University of Florida, Gainesville, Florida, United States of America; 2 Florida Museum of Natural History, University of Florida, Gainesville, Florida, United States of America; 3 School of Life Sciences, Arizona State University, Tempe, Arizona, United States of America; 4 School of Biological Sciences University of Auckland, Auckland, New Zealand; CNRS, FRANCE

## Abstract

Male courtship display is common in many animals; in some cases, males engage in courtship indiscriminately, spending significant time and energy courting heterospecifics with whom they have no chance of mating or producing viable offspring. Due to high costs and few if any benefits, we might expect mechanisms to evolve to reduce such misdirected courtship (or ‘reproductive interference’). In *Habronattus* jumping spiders, males frequently court heterospecifics with whom they do not mate or hybridize; females are larger and are voracious predators, posing a severe risk to males who court indiscriminately. In this study, we examined patterns of misdirected courtship in a natural community of four sympatric *Habronattus* species (*H*. *clypeatus*, *H*. *hallani*, *H*. *hirsutus*, and *H*. *pyrrithrix*). We used direct field observations to weigh support for two hypotheses (differential microhabitat use and species recognition signaling) to explain how these species reduce the costs associated with misdirected courtship. We show that, while the four species of *Habronattus* do show some differences in microhabitat use, all four species still overlap substantially, and in three of the four species individuals equally encountered heterospecifics and conspecifics. Males courted females at every opportunity, regardless of species, and in some cases, this led to aggression and predation by the female. These results suggest that, while differences in microhabitat use might reduce misdirected courtship to some extent, co-existence of these four species may be possible due to complex communication (i.e. species-specific elements of a male’s courtship display). This study is the first to examine misdirected courtship in jumping spiders. Studies of misdirected courtship and its consequences in the field are limited and may broaden our understanding of how biodiversity is maintained within a community.

## Introduction

In many animals, courtship displays have evolved to facilitate successful mating, often by providing information about a potential mate’s location, sex, species, or quality as a mate (reviewed in [1]). Yet, courtship often incurs costs, such as increased energy expenditure (e.g., [2–4]), decreased longevity (e.g., [5–6]), and increased predation risk (e.g., [7–8]). We would thus expect that selection should favor individuals that reduce their courtship efforts in situations where these costs outweigh potential reproductive benefits. However, many animals (e.g., ground-hoppers, moths, flies, ticks, lizards, fish) invest time and energy courting heterospecifics with which they never mate or are unable to produce viable offspring (reviewed in [9]), or even attempt mating with inanimate objects (e.g., [10]). In addition to simply wasting energy that could be invested in other activities, such misdirected courtship (or ‘reproductive interference’) can also reduce or prevent viable mating opportunities for both sexes (e.g., [11–16]). Given such costs, we might expect selection to favor mechanisms that prevent or reduce misdirected courtship. However, this topic has been given little attention in the ecological literature and is strongly biased towards laboratory rather than field studies where the ecological relevance is sometimes unclear (see [9]).

Jumping spiders (family Salticidae) are an excellent group in which to examine mechanisms that reduce heterospecific courtship because, for male jumping spiders, the consequences of courting a female of the wrong species can be severe. Females of most salticid species are generalist predators and thus courting males, even of the same species, can become either a potential mate or prey item; as such, cannibalism from conspecific females and predation from heterospecific females is an important risk (reviewed in [17]). Even *conspecific* courtship is risky; if heterospecific courtship never results in offspring, we might expect strong selection on males to avoid it. Despite a growing body of literature addressing misdirected courtship in animals, few studies have considered the potential cost of predation from the female that is being courted (see [9] for a review of misdirected courtship); this cost is unique to voracious and cannibalistic predators such as spiders [12] and praying mantises [13].

Across the jumping spider genus *Habronattus*, adult males are not very discriminating in courtship; in the lab, they readily court dead conspecific female specimens as well as live heterospecific females (LAT, unpub. data). Such indiscriminate male courtship behavior may be responsible, in part, for driving the patterns of hybridization seen among some *Habronattus* species that overlap in the field [18–19]. It may not be surprising if males cannot easily visually discriminate among females as they are relatively drab and similar to one another in coloration and do not engage in overt receptivity displays to courting males (LAT, unpub. data). Here we examine patterns of heterospecific courtship under natural conditions where multiple *Habronattus* co-occur. Specifically, we worked in a riparian area in which four sympatric species of *Habronattus* exist in high abundance and overlap in the timing of sexual maturity and mating. These particular species are all from different species groups [18] and do not hybridize (LAT, pers. obs.), yet males from all four species have been observed to readily court females of any of the other species in the lab, even though both conspecific and heterospecific adult females are voracious and cannibalistic predators [20]. Female predation on adult males (both conspecific and heterospecific) has been documented on numerous occasions in both the lab and field ([20], LAT, pers. obs.), although the extent to which it is occurs across different *Habronattus* species has not been studied. In addition to the risks of predation from females, courtship for *Habronattus* males is likely to be energetically costly; males engage in dramatic dances consisting of coordinated combinations of color, motion, and seismic cues (e.g., [18, 21–25]) and will court continuously for hours in the lab, even if females are unreceptive or aggressive (LAT, pers. obs). In addition to the energetic expense of dancing, the conspicuously colored ornaments that males display to females (e.g., [26–27]) may increase predation risk by visual predators (see [28]).

**Fig 1:**
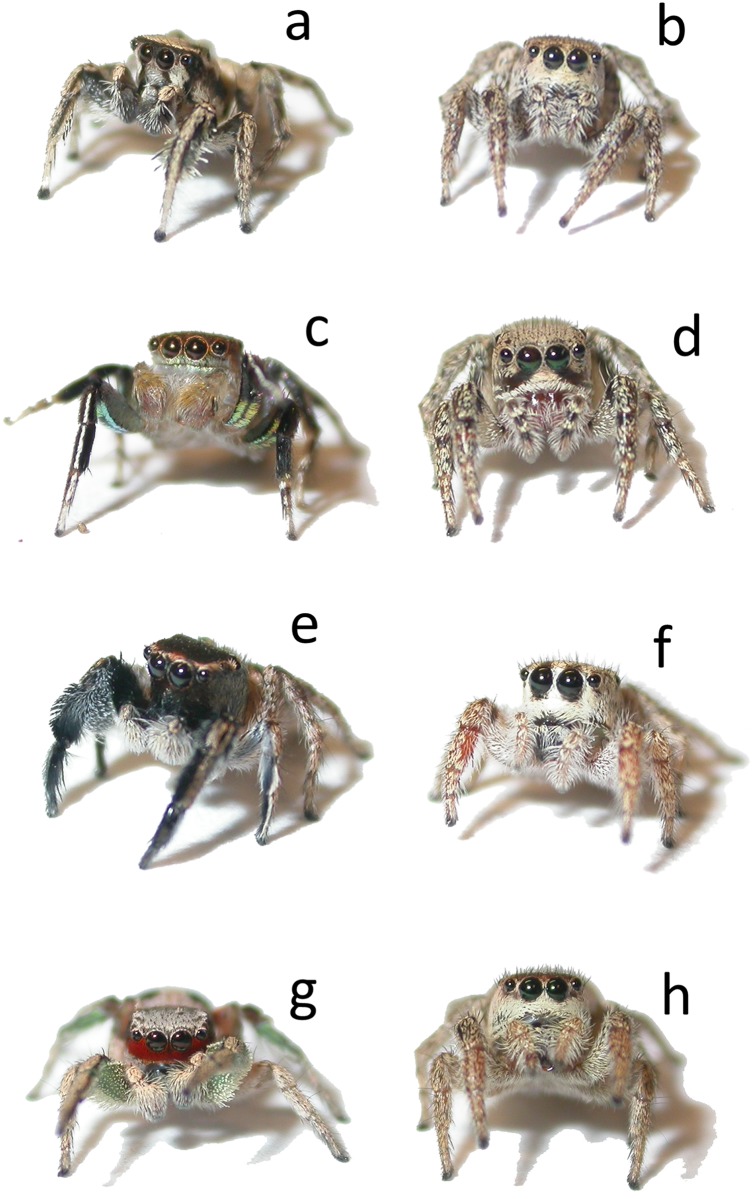
Adult sexual dichromatism on the face and legs of four sympatric species of *Habronattus*. *H*. *clypeatus* male (a) and female (b), *H*. *hallani* male (c) and female (d), *H*. *hirsutus* male (e) and female (f), and *H*. *pyrrithrix* male (g) and female (h). Though females all look similar to one another, they can be identified based on subtle differences in dorsal and facial markings.

In this study, we aim to address the following question: under natural conditions, how do males reduce or avoid high costs associated with misdirected heterospecific courtship? Specifically, we use direct observations of spiders in the field to weigh support for two potential hypotheses for the reduction of heterospecific courtship costs. Our first hypothesis is that heterospecific interaction rates are effectively reduced by differential use of the microhabitat (e.g., substrate, light environment) by the four species. Field observations of *Habronattus* indicate that females spend much of their time at rest in particular microhabitats (for feeding, nesting, etc.) while males spend much of their time actively moving, seeking out, and courting females at every opportunity [20]. Thus, if the *Habronattus* species in our study are utilizing the habitat differently, we expect females of the four species to be partitioned in space while males move around in areas where they would be most likely to find conspecific females. In each of the four species in our study, males have very different colorful ornaments, ranging in color from solid black, to black and white striped, to bright red, to iridescent green and pink (see Fig 1, see also [29]). In addition, males of these species also differ dramatically in vibratory aspects of their display; one produces no vibrations, one produces simple low-frequency vibrations, and two produce highly complex vibratory displays (D. Elias, personal communication). Because color signal transmission is strongly affected by both the visual background and the light environment where courtship takes place (e.g., [30–33]) and vibratory transmission is strongly affected by physical properties of the substrate [34], it is reasonable to expect differences in substrate preferences of females from these different species, in order to optimize perception of their conspecific male’s particular display. If the four species are indeed partitioned in space with little overlap, this may explain why males have adopted the strategy of indiscriminately courting every female they encounter; such a strategy may be beneficial if heterospecific interactions are relatively rare compared with conspecific interactions.

An alternative hypothesis is that the four species do not differ in microhabitat use; as a result, heterospecific interactions will be just as common as conspecific interactions. Such high heterospecific interaction rates would mean that males and females must rely solely on communication with one another to identify appropriate, conspecific, mates. Animals may rely on multiple signals to achieve positive species recognition [35], some of which begin from afar and become more subtle (and species-specific) as the conspecific approaches [36–37]. If this is the case, it may help to explain why *Habronattus* males have evolved such a rich diversity of multi-modal display traits, as these may be necessary to capture female attention and subsequently communicate species identity to females from a safe distance as they approach.

In this study, we use focal behavioral observations on free-ranging spiders to weigh support for the differential-microhabitat-use and species-recognition-signaling hypotheses. While we present them as discrete alternatives, both generating clear and testable predictions, we anticipate that subtleties in the data will allow us to weigh the relative support for each hypothesis. Ultimately, we will be able to place each species along a hypothetical continuum ranging from complete isolation from others (i.e., heterospecifics do not interact in the field) to complete overlap (i.e., heterospecific interaction rates are as common as conspecific interaction rates). This study is the first to examine misdirected courtship in jumping spiders, a ubiquitous and diverse taxon with more than 5000 species worldwide [38]. A better understanding of how jumping spider species overlap and interact under natural conditions will help explain how similar species can co-exist, allowing for the maintenance of biodiversity within a habitat.

## Methods

### Study species

The genus *Habronattus* is the most diverse jumping spider genus, with approximately 100 species [18]. Males and females have trichromatic vision that enables color discrimination from UV through red [39], and males are highly ornamented with a striking diversity of brilliantly colored faces and legs that they display to drab, cryptic females during elaborate courtship dances (e.g., [18, 21, 23–25, 40]). When a *Habronattus* male locates a female in the field, he begins his display with a species-specific combination of leg-waving motions and then often zig-zags or sidles back and forth as he slowly approaches her (LAT, pers. obs). In addition to these visual aspects of display, males of many species also produce species-specific substrate-borne vibrations as part of their display, particularly as they advance through courtship and get closer to females [41]. Evidence from geographically isolated sky island populations of *Habronattus pugilis* suggests that sexual selection is responsible for driving such striking male diversification [19, 42].

Our study focused on four sympatric *Habronattus* species, described in more detail below. There is currently no field data available on the daily activity patterns or reproductive periods of these four species. At our field site, individuals of all ages and both sexes are active throughout the day (LAT, pers. obs.). Adult males and females can be found year-round, but are most commonly encountered between March and November (LAT, pers. obs.). Adult females of all four species are voracious generalist predators and are generally larger than adult males; predation by females on both conspecific and heterospecific males has been documented on numerous occasions ([20], LAT, pers. obs.). Geographic variation in coloration is common within the genus (see [29]) and thus some subtleties of the color pattern described here might be typical of this population in Phoenix, Arizona, USA.

#### *Habronattus clypeatus* (Banks)

Adult male *H*. *clypeatus* have white faces with contrasting dark vertical bands beneath their anterior median eyes, and the undersides of their first pair of legs used in display are gray and covered with white, spatulate scales (Fig 1a). In addition to their visual displays, males also produce complex vibratory songs that increase in complexity and intensity as they approach females (D. Elias, personal communication). Females are a drab gray and brown with white faces (Fig 1b). *H*. *clypeatus* is found in northern Mexico and the southwestern USA and as far north as Wyoming, USA [29].

#### *Habronattus hallani* (Richman)

In adult male *H*. *hallani*, the faces and first two pairs of legs are adorned with iridescent scales that change in hue from green to pink, depending on viewing angle (Fig 1c). Unlike many other Habronattus species, *H*. *hallani* males do not have a vibratory component to their display (D. Elias, personal communication). Females are a drab gray and brown with white faces and characteristic dark, curved bands below their anterior median eyes (Fig 1d). *H*. *hallani* is distributed through the southwest USA to northern Mexico [29].

#### *Habronattus hirsutus* (Peckham and Peckham)

Adult male *H*. *hirsutus* have dark gray/black front legs, the underside of which exhibit a narrow greenish band, and are further adorned with dense hairs (Fig 1e). Most adult males in our focal population have completely black faces (Fig 1e), but we have observed occasional males with bright red facial patches (LAT, pers. obs., see S1 Fig). This degree of variation in facial coloration is typical on a geographic scale but is not well understood (see [29]). More than 95% of males in our focal population were of the black-faced form, including all of those that were the subjects of focal observations. In addition to their visual displays, male *H*. *hirsutus* produce simple, low-frequency vibrations during courtship (D. Elias, personal communication). Females are a drab gray and brown with white faces that have subtle dark markings just below and just above the anterior median eyes (Fig 1f). *H*. *hirsutus* is broadly distributed across western North America, from southern Canada to Mexico [29].

#### *Habronattus pyrrithrix* (Chamberlin)

Adult male *H*. *pyrrithrix* have bright red faces and green front legs (Fig 1g), both of which are condition-dependent and displayed to females during courtship [26–27]. The presence of male red facial coloration improves courtship success when males are courting in the sunlight [43]. Similar to *H*. *clypeatus* (described above), male *H*. *pyrrithrix* produce highly complex vibratory songs along with their visual displays [41]. Females are drab gray and brown with white faces (Fig 1h). *H*. *pyrrithrix* is distributed from the southwest USA to Sinaloa, Mexico [29].

### Study site

All behavioral observations were made at the Rio Salado Habitat Restoration Area (RSHRA) in Phoenix, Arizona, (Maricopa County, 33.42°N, 112.07°W), USA. The purpose of the RSHRA is to reestablish native wetland and riparian habitats that were historically associated with the Salt River (Rio Salado), which used to flow year-round [44]. *Habronattus* were generally concentrated in the leaf litter and vegetation within the gallery forests dominated by cottonwood (*Populus fremontii*) and desert willow (*Chilopsis linearis*). Permission to conduct this work was granted by the City of Phoenix Parks and Recreation Department.

### Data collection

Behavioral observations were carried out between 900 and 1500 hrs. from March to November in 2009 and 2010. We located *Habronattus* by visually scanning the leaf litter and vegetation in the field. When we located a spider, we conducted a 15-minute behavioral observation in which we followed that spider from approximately 1m away and recorded behavior using voice recorders. Our sample sizes vary due to differences in abundance among species (*H*. *clypeatus*: n = 12 (5 females, 7 males), *H*. *hallani*: n = 14 (8 females, 6 males), *H*. *pyrrithrix*: n = 34 (20 females, 14 males), *H*. *hirsutus*: n = 27 (10 females, 17 males)).

We tracked every transition spiders made between sunlight and shade and used this to quantify the total amount of time spent in each. We also quantified the amount of time spent on different substrate types (cottonwood leaf litter, desert willow leaf litter, cottonwood vegetation, desert willow vegetation, grass, or dirt/rock). We recorded all interactions between the focal spider and either conspecific or heterospecific *Habronattus*. We defined an ‘interaction’ as any case in which both spiders responded to the presence of the other by orienting their anterior median eyes at the other individual (e.g., [45]). We recorded all instances of courtship, aggression (attacks), predation/cannibalism, and copulation. Because we frequently saw other individuals in the vicinity that did not interact with the focal spider, but that still provided valuable information about the local abundance and activity of the community, we recorded the number of other non-interacting *Habronattus* that we saw within 0.5m of the focal spider during the observation. Because our attention was focused on the behavior of the focal individual, our estimates of other *Habronattus* in the vicinity are likely to be more conservative than the actual abundance of spiders inhabiting the area. If spiders captured prey or were found feeding during the focal observation, we recorded the identity of the prey item (identified to family when possible).

After behavioral observations were completed, we temporarily captured each individual in a clear plastic vial. We confirmed the maturity of females by examining their epigynum; mature females can be distinguished from immatures by the presence of a sclerotized epigynum [46]. To ensure that no individual was observed more than once, we marked spiders after observations with a small black dot (~1mm in diameter) on the underside of their abdomen using non-toxic liquid eyeliner (Urban Decay Cosmetics, Costa Mesa, CA, USA), which produced a permanent mark.

### Data analysis

To determine if females of the four species utilized the available microhabitat differently, we compared substrate and light environment use (% of time spend on the different substrates and % of time spent in the sun and shade) among females of the four species using nonparametric Kruskal-Wallis tests with Steel-Dwass pairwise comparisons (α = 0.05). For substrate, we first compared each species’ use of the three broad categories of microhabitat (leaf litter, vegetation, dirt/rock) and then we repeated the analysis on a finer scale that considered more subtle differences in microhabitat (cottonwood leaf litter, willow leaf litter, cottonwood vegetation, willow vegetation, grass, dirt/rock). We then compared the percentage of time that females of each species spent in the sun (vs. the shade).

To examine if males were located in microhabitats where they would be most likely to encounter conspecific females, we then determined if there was a correlation across species between female microhabitat use (i.e., substrate, light environment) and male microhabitat use using non-parametric Spearman rank correlations. Because there was a clear difference in broad patterns of habitat use, with *H*. *hirsutus* spending the majority of time in the vegetation and the other three species spending the majority of time in the leaf litter (see Results), we ran an additional analysis on just the litter-dwelling species to determine if males of these three species were preferentially found in microhabitats where they would be most likely to find conspecifics.

To compare the mean number of conspecific interactions during focal observations with the number of heterospecific interactions for each species, we used non-parametric Wilcoxon signed rank tests. Because our study focused on the ecological importance of misdirected courtship, we conducted a second analysis, where we excluded interactions with juveniles and only examined interactions between sexually mature adults.

We used non-parametric statistics because our data did not meet relevant assumptions. All statistical analyses were performed using SAS 9.2 and JMP 9.0.2 (SAS Institute, Cary, NC, USA).

## Results

### Use of substrate and light environment

**Fig 2:**
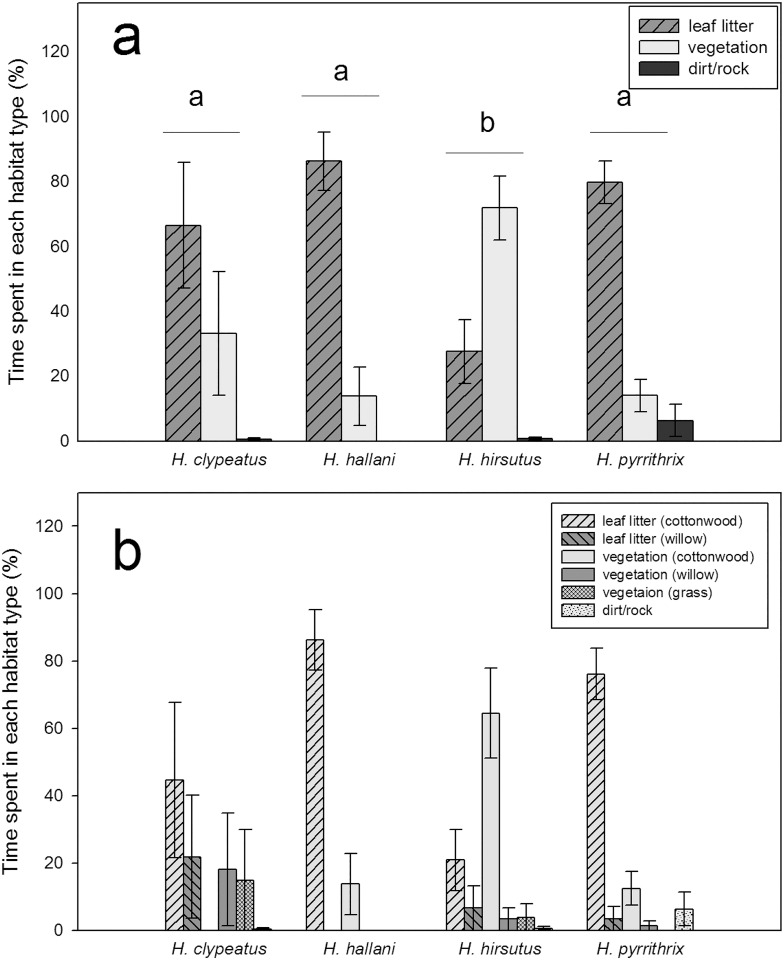
Comparison of the percentage of time that females of each species spent on different (a) broad categories of substrate and (b) finer categories of substrate during field behavioral observations (mean ± SEM). In (a), different letters indicate significant differences between species in their use of the leaf litter and vegetation.

**Fig 3:**
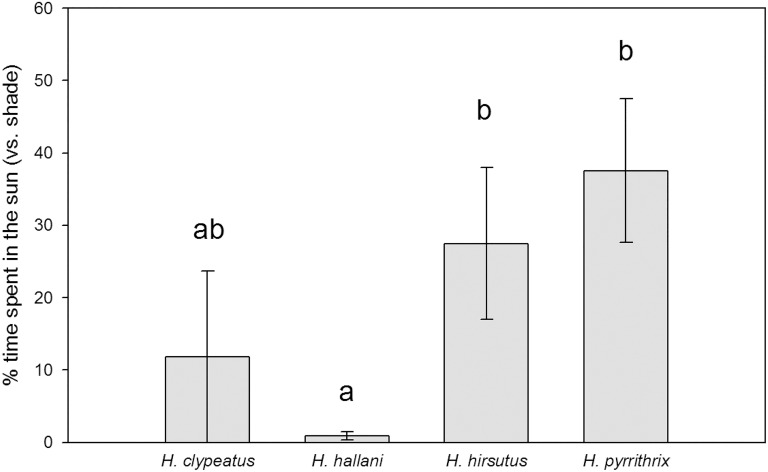
Comparison of the percentage of time that females of each species spent in the sun during behavioral observations in the field (mean ± SEM). Unshared letters indicate significant differences between species.

Female *H*. *clypeatus*, *H*. *hallani*, and *H*. *pyrrithrix* all spent the majority of their time on the ground in the leaf litter (67%, 86%, and 80% of their time, respectively). In contrast, female *H*. *hirsutus* spent the majority of their time above the ground in the vegetation (72%). While there were significant differences among species in the females’ use of the leaf litter and vegetation (leaf litter: *X*^2^ = 15.29, *P* = 0.0016, vegetation: *X*^2^ = 16.85, *P* = 0.0008; dirt/rock: *X*^2^ = 2.69, *P* = 0.441), there was still substantial overlap; females of all four species were found, at least occasionally, in both the leaf litter and the vegetation (Fig 2a). When we examined female substrate use on a finer scale, we again found significant differences among the species in their use of the substrate, but again there was substantial interspecific overlap in substrate use (cottonwood leaf litter: *X*^2^ = 14.86, *P* = 0.0019, willow leaf litter: *X*^2^ = 6.45, *P* = 0.084, cottonwood vegetation: *X*^2^ = 14.44, *P* = 0.0024, willow vegetation: *X*^2^ = 6.70, *P* = 0.082; grass: *X*^2^ = 4.66, *P* = 0.198, dirt/rock: *X*^2^ = 2.69, *P* = 0.441, Fig 2b). Females of the four species differed in the amount of time spent in the sunlight, with *H*. *hallani* spending the least time in the sunlight and *H*. *hirsutus* and *H*. *pyrrithrix* spending the most (*X*^2^ = 10.80, *P* = 0.013; Fig 3).

When all four species were analyzed together, there were significant positive correlations between female substrate location and the location of conspecific males in some, but not all, substrate types (Table 1). Similarly, when the analysis was restricted to the three predominantly litter-dwelling species (*H*. *clypeatus*, *H*. *hallani*, and *H*. *pyrrithrix*), there were significant positive correlations between the location of females and conspecific males in some, but not all, substrate types (Table 2). The amount of time females spent in the sunlight was not correlated with the amount of time that conspecific males spent in the sunlight, either when all four species were analyzed together (Table 1) or when the analysis was restricted to the predominantly litter-dwelling species (Table 2).

**Table 1:** Spearman rank correlations between female habitat preference and the preference of conspecific males in four species of sympatric *Habronattus* (*H*. *clypeatus*, *H*. *hallani*, *H*. *hirsutus*, and *H*. *pyrrithrix*). Significant correlations suggest that males may be searching for females in specific habitats where they may be most likely to find conspecifics.

Microhabitat	Rho (ρ)	*P*
Light environment		
Sunlight (vs. shade)	0.400	0.600
Broad substrate category		
Leaf litter	1.000	<0.001
Vegetation	1.000	<0.001
Dirt/rock	0.770	0.225
Finer scale substrate categories		
Cottonwood* leaf litter	1.000	<0.001
Willow† leaf litter	0.632	0.368
Cottonwood* vegetation	0.316	0.684
Willow† vegetation	0.800	0.200
Grass	-0.544	0.456

* Populus fremontii

^†^ Chilopsis linearis

**Table 2:** Spearman rank correlations between female habitat preference and the preference of conspecific males in three litter-dwelling species of *Habronattus* (*H*. *clypeatus*, *H*. *hallani*, and *H*. *pyrrithrix*). Note that the primarily vegetation-dwelling *H*. *hirsutus* is excluded from this analysis. Significant correlations suggest that males may be searching for females in specific habitats where they may be most likely to find conspecifics.

Microhabitat	Rho (ρ)	*P*
Light environment		
Sunlight (vs. shade)	0.500	0.667
Broad substrate category		
Leaf litter	1.000	<0.001
Vegetation	1.000	<0.001
Dirt/rock	0.867	0.333
Finer scale substrate categories		
Cottonwood* leaf litter	1.000	<0.001
Willow† leaf litter	1.000	<0.001
Cottonwood* vegetation	-0.867	0.333
Willow† vegetation	1.000	<0.001
Grass	-0.500	0.667

* Populus fremontii

^†^ Chilopsis linearis

### Behavioral interactions

**Fig 4:**
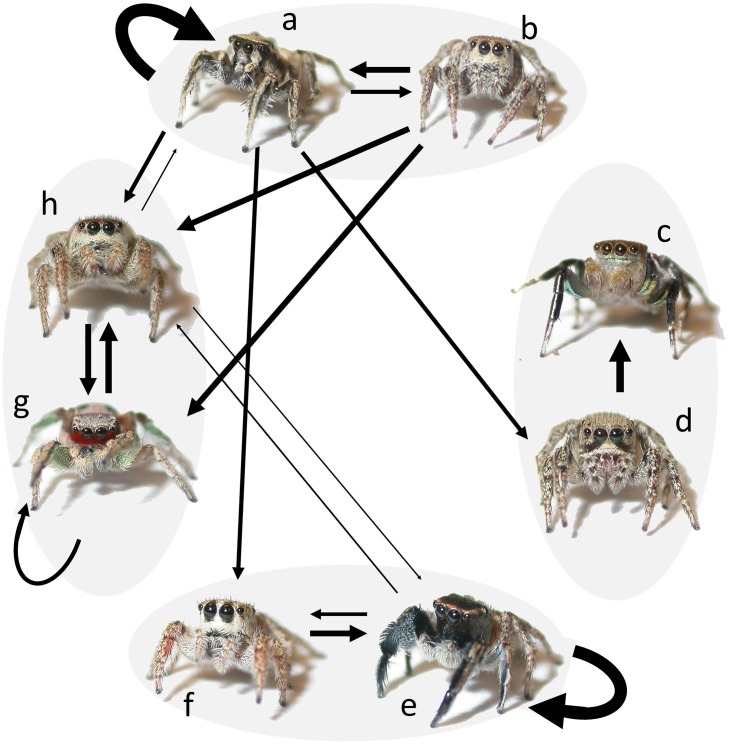
Summary of interactions between sexually mature adults in four species of sympatric *Habronattus* illustrating how rates of misdirected courtship likely vary by species. Thickness of arrows represents the relative frequency of interactions per observation (with wider arrows indicating more frequent interactions). Curved arrows indicate interactions between individuals of the same species and sex. In 100% of interactions between adult males and adult females (regardless of species), courtship occurred. *H*. *clypeatus* male (a) and female (b), *H*. *hallani* male (c) and female (d), *H*. *hirsutus* male (e) and female (f), and *H*. *pyrrithrix* male (g) and female (h).

Densities of *Habronattus* were high; in 57 of 87 (66%) observations we spotted at least one other *Habronattus* within a 0.5m radius of the focal individual and in 33 (38%) observations, the focal spider interacted with at least one other *Habronattus*. We observed a total of 42 interactions between focal spiders and other *Habronattus*, 35 (83%) of which occurred between sexually mature adults (see specific breakdown of interactions among species and sexes in Fig 4). Of the interactions between sexually-mature adults, twenty-seven (77%) of these involved interactions between conspecifics and 8 (23%) involved interactions between heterospecifics. Twenty-two (52%) of all interactions occurred between sexually mature males and females (15 of which were between conspecifics and 7 between heterospecifics). In 100% of these 22 interactions, regardless of whether or not they were conspecifics or heterospecifics, males engaged in courtship. During courtship interactions, males were attacked in four cases (18%); 3 of these occurred during conspecific courtship (between male and female *H*. *pyrrithrix*) and one during heterospecific courtship (between a male *H*. *clypeatus* and a female *H*. *pyrrithrix*). In one of the cases of conspecific aggression, the male was attacked several times by the female, but he continued to court and eventually copulated with her; copulation occurred in the leaf litter in full sunlight. In the heterospecific case, the male was attacked several times and was eventually eaten by the female. One instance of aggression was observed between adult females, when an *H*. *clypeatus* attacked (but did not kill) a female *H*. *pyrrithrix*. No aggression was observed between males.

**Fig 5:**
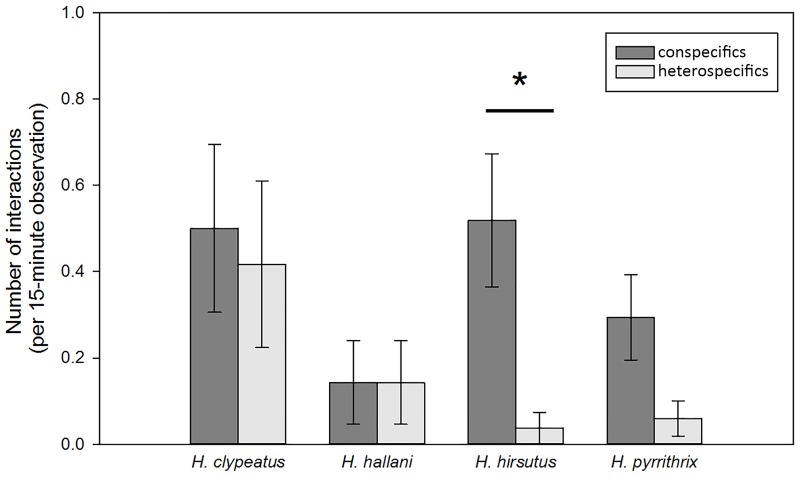
Comparison of the number of conspecific and heterospecific interactions during behavioral observations in the field (mean ± SEM). Asterisks (*) indicate significant differences between conspecific and heterospecific interaction rates within a species.

For *H*. *clypeatus*, *H*. *hallani*, and *H*. *pyrrithrix*, there were no significant differences between conspecific and heterospecific interaction rates (i.e., an individual was just as likely to interact with a heterospecific as they were with a conspecific, although *H*. *pyrrithrix* tended to have more conspecific than heterospecific interactions; *H*. *clypeatus*: *S* = -2.00, *P*>0.999; *H*. *hallani*: *S* = 0.00, *P*>0.999; *H*. *pyrrithrix*: *S* = -90.5, *P* = 0.051; Fig 5). For *H*. *hirsutus*, individuals had significantly more interactions with conspecifics than heterospecifics (*S* = -100.5, *P* = 0.004; Fig 5). When we limited our analysis to interactions between sexually mature adults (excluding interaction with juveniles), there were again no significant differences between conspecific and heterospecific interaction rates in *H*. *clypeatus*, *H*. *hallani*, or *H*. *pyrrithrix*, although *H*. *pyrrithrix* tended to have more conspecific than heterospecific interactions (*H*. *clypeatus*: *S* = 0.00, *P* = 1.00; *H*. *hallani*: *S* = -13.50, *P* = 0.50; *H*. *pyrrithrix*: *S* = -77.0, *P* = 0.088). Again, in *H*. *hirsutus*, individuals had more interactions with conspecifics than heterospecifics (*S* = -72.0, *P* = 0.023).

To compare these interaction rates with other ecologically relevant events, in only 2 of 87 focal observations (2%) did we see the focal individual capture prey. In both cases, the focal spider was a female *H*. *hallani* attacking and eating a juvenile *H*. *hirsutus* (~3mm in size).

## Discussion

In this study, we examined two hypotheses to explain how four sympatric *Habronattus* jumping spider species might avoid the high costs associated with heterospecific courtship in the field. Our results suggest that, while there is some evidence that the four species utilize the habitat and light environment differently, there is still substantial overlap between all four species. This overlap leads to high interaction rates among species and high rates of heterospecific courtship, suggesting that aspects of communication likely play a role in mitigating the costs of these interactions.

In other species where reproductive interference is costly, habitat partitioning has been suggested as a mechanism that allows species to co-exist (e.g., [47]). In this study, we show that females of four different species of *Habronattus* do indeed utilize the available microhabitats and light environments slightly differently, which may reduce heterospecific interactions to some extent. Specifically, female *H*. *hirsutus* spend much of their time above the ground in the vegetation, while the other three species are predominantly ground-dwelling. Among the ground dwellers, all three species spent most of their time in cottonwood leaf litter, substantially overlapping in the type of habitat used. The four species also showed different light environment use, with *H*. *hallani* females spending the least time in the sun and *H*. *hirsutus* and *H*. *pyrrithrix* spending the most. Our study relied on tracking focal individuals and observing behavior in detail, rather than systematically sampling every individual in different substrates at different times of the day or across seasons and years. One limitation of our approach is that we can’t rule out the possibility that our focal observations were biased towards habitats where spiders were more obvious. Additional systematic sampling (e.g., exhaustively sampling every individual present along transects at regular intervals throughout the year) will be an important next step to more fully characterize patterns of habitat partitioning.

Because females generally spend most of their time at rest, while males spend most of their time moving (e.g. actively searching for females [20]), we went on to test if males searched for females in microhabitats where they would be most likely to find conspecifics, rather than heterospecifics. Our results indicate that there were significant positive correlations between female substrate use and the substrate use of conspecific males in some, but not all, substrate types. This suggests that, in some cases, males may be searching for conspecific females on substrates where they are most likely to find them. However, there was no correlation between the light environment locations of males and females across species, suggesting that males are likely not biasing their mate search towards light environments where they are most likely to find conspecific females.

An alternative explanation for how *Habronattus* species might mitigate the high costs of heterospecific courtship is that, rather than being environmentally segregated, they simply rely on communication with every individual that they encounter to identify appropriate courtship/mating targets. While we found some evidence of microhabitat partitioning, we also found very high rates of interaction among all four species, suggesting that communication is likely important in reducing the costs of misdirected courtship. To put these interaction rates in perspective, over the course of 87 focal observations (15 minutes each), we observed only two focal spiders capturing prey, but we observed 42 focal spiders interacting with other individuals. Of those interactions, 22 involved courtship. For each spider, this is approximately 2 interactions per hour (or 1 courtship interaction per hour), compared with only 0.092 prey items captured per hour. Interactions with other individuals are clearly common and ecologically relevant events for these spiders. Interestingly, for three out of the four species (*H*. *clypeatus*, *H*. *hallani*, and *H*. *pyrrithrix*), individuals were just as likely to interact with a heterospecific as they were with a conspecific. Not surprisingly, *H*. *hirsutus* was the only species where conspecific interactions were significantly more likely than heterospecific interactions; this is likely because *H*. *hirsutus* spends most of its time in the vegetation, away from the other three species. While misdirected heterospecific courtship was the focus of this study, the high rates of conspecific courtship were also notable, suggesting that females likely have the opportunity to be choosy and males likely have the opportunity to mate multiple times. Similarly, in a recent field study of *Habronattus americanus*, females encountered approximately one courting conspecific male per hour but rejected most, indicating high levels of female choosiness [48]. Clearly, frequent communication both within and between species is an important aspect of *Habronattus* ecology.

For male animals that provide no resources to their mate (e.g., food, parental care), selection may favor those that mate multiply and indiscriminately [49]. However, for male *Habronattus*, indiscriminate courtship comes with a risk that is not faced by males in many other taxa: female aggression and predation. While this phenomenon has not been well-studied, there is recent evidence from two taxa of voracious predators (praying mantises and gift-giving spiders) that indiscriminate courtship by males leads to aggression and predation by heterospecific females [12–13]. Our data show that female aggression in *Habronattus* can occur during both conspecific and heterospecific courtship in the field. Attacks on courting males occurred in 18% of our courtship observations (one which resulted in the male being killed). Given how much of their time males spend courting and that courtship can last for hours, and extend well beyond our 15-minute focal observation period (LAT, pers. obs.), attacks from females are likely to be a significant and ecologically-relevant risk for males in the field. In conspecific courtship, it is possible that this risk is outweighed by the possibility of successful copulation; before succeeding, the only focal male in our study that copulated was first attacked several times by the same female. However, in the case of heterospecific courtship, males are unlikely to gain any benefit from courting a heterospecific, but they pay the same cost; one male in our study was attacked several times by a heterospecific female who eventually captured and ate him. There is growing evidence in spiders that courtship displays incur both energetic and viability costs for males, as well as increased risk of predation [4, 8, 16, 28, 50–53]. However, we argue that what makes spiders a particularly intriguing system to examine misdirected courtship is the risk of predation from females, a cost that is only rarely examined in this context (see review in [9]).

Females may also incur costs associated with courtship; in some species of jumping spiders, evidence suggests that females actually face a higher predation risk than the males who are courting them [54]. In water striders, males of some species court and attempt matings with females indiscriminately [55]. Females often struggle to deter or dislodge males that are attempting to copulate with them, but such struggling results in a 200% increase in energy expenditure [56]. Female *Habronattus* also likely pay a similar cost for misdirected courtship. In this study, courting males who were rejected in the field often pursued females, even when they attacked males or tried to hop away. Given the high heterospecific interaction rates observed in our study, constantly moving away from courting heterospecific males is likely to incur energetic costs for females and increase their conspicuousness to predators. While females are often bigger than males and can readily attack them, we provide observational data in the field showing that initial attacks are not always successful and that males may dodge attacks while continuing to court. All of these observations suggest that misdirected courtship likely incurs costs for female *Habronattus*, as well as males.

It seems likely that the colorful, species-specific ornaments and multimodal courtship displays of male *Habronattus* help them reduce the risks associated with misdirected courtship. If these displays allow a male to identify himself from a distance, indiscriminate courtship may give him the opportunity to safely assess a female’s receptivity or aggression. Work done with two other jumping spider species (*Cosmophasis umbratica* and *Phintella vittata*) has shown that blocking UV light affects mate-choice decisions, suggesting that UV coloration plays an important role in sex and/or species recognition in these species [57–59]. In *Habronattus* however, the role of color as a species recognition signal is less straightforward. In *H*. *pyrrithrix*, the presence of a male’s bright red face and green leg coloration are not required for successful copulation, although the presence of red facial coloration improves male courtship success only in certain contexts (i.e. under bright lighting conditions [43]). Although not a required species recognition signal, elaborate male colors may help females assess a male’s species identity under certain environmental conditions. If a male’s display colors help him to signal and assess female receptivity from a safe distance, then he may use additional signal components to provide more information as he approaches. In many *Habronattus* species, complex, species-specific vibratory signals are incorporated into the courtship display and appear to be important only after males have moved closer to females [24, 41]. Additionally, other species of jumping spiders rely heavily on chemical signals in mate choice (e.g., [60–61]); it is possible that *Habronattus* displays are just as complex in this modality but have yet to be examined. More work is clearly needed to examine how male *Habronattus* displays may enable recognition and avoid aggression by females.

In the present study, the finding that females of the four species of *Habronattus* utilized the available substrate and light environment differently is consistent with the idea that male species-specific colors have been selected to maximize signal transmission when communicating with conspecific females in different light environments (e.g., [33]). Male coloration is geographically variable in many species (see [29]), suggesting that color patterns may be locally adapted to specific attributes of their environment. While this hypothesis should be examined on a larger phylogenetic scale, results from the present study provide some intriguing patterns to be investigated further. First, female *H*. *hallani* spent the least time in the sun (see Fig 3); males of this species have iridescent markings (see Fig 1c) that might allow them to maximize signal transmission under low light levels, where other colors are less effective (e.g., red, see [43]). In contrast, female *H*. *pyrrithrix* spent the most time in the sun. As described above, the bright red face of male *H*. *pyrrithrix* only improves male courtship success in the sun (but not in the shade), presumably due to the fact that sunlight is richer in red light while forest and woodland shade is relatively low in red light [32]. The fact that *H*. *hirsutus* also spends most of their time in the sun may not appear to fit this pattern, yet some males in this population do indeed exhibit red coloration on their faces (see S1 Fig) and across their geographic range, red facial coloration in *H*. *hirsutus* is relatively common [29]. Clearly, these qualitative relationships are preliminary and speculative but warrant further study within a larger, phylogenetically-controlled framework. Future work should also consider how each species’ display colors contrast with the visual backgrounds of different habitats.

Similarly, male species-specific vibratory displays may also be well-matched to the physical attributes of their microhabitats in ways that maximize signal transmission (as hypothesized in [34]). Specifically, both *H*. *clypeatus* and *H*. *pyrrithrix* have complex vibratory components in their courtship displays (D. Elias, personal communication). Work with *H*. *dossenus* (a species with similarly complex displays) has shown that leaf litter transmits such vibrations with the least attenuation and females are more likely to mate with males who court on leaf litter (compared with sand or rocks) [34]. As expected, our data showed that both *H*. *clypeatus* and *H*. *pyrrithrix* were most commonly found in leaf litter (compared with all other substrate types). In contrast, *H*. *hirsutus* displays have only simple low-frequency songs; while speculative, it may be that such vibrations are better matched to the cottonwood vegetation (i.e., twigs and branches) which transmit low-frequency sound well (D. Elias, personal communication). Finally, *H*. *hallani* do not use vibrations in their display (D. Elias, personal communication) and instead have one of the most elaborately colored iridescent displays (see Fig 1); as such, their microhabitat choice may be more tied to visual (rather than vibratory) aspects of their display. Again, such patterns are speculative at this stage but certainly warrant further study.

The costs of misdirected courtship and heterospecific mating attempts are often density-dependent and may affect interacting species in different ways depending on their relative abundance (e.g., [62]). Interestingly, in the community of *Habronattus* examined in our study, *H*. *hallani* is the least abundant species of the four across five years of observations (LAT, unpublished data) and is also generally found in low abundance in other areas of its range (LAT, pers. obs.). As such, we might expect *H*. *hallani* females to incur higher relative costs due to misdirected courtship than the other species, and thus females might benefit from additional mechanisms of signaling species identity to males. Interestingly, of the three species, *H*. *hallani* is the only species where females exhibit striking facial patterns (see Fig 1d). Future studies should examine the roles of female face markings within a larger phylogenetic framework to test the idea that they are more likely to evolve in situations where the costs of misdirected courtship are highest. Field studies that systematically examine patterns of relative abundance of these four species across space and time may help us better understand patterns of reproductive interference and predict where it is most likely to occur.

A recent review of the literature on misdirected courtship found a strong bias towards laboratory studies (n = 27) compared with field experiments (n = 8) and field observations (n = 9) [9]. Groening and Hochkirch [9] stress the limitations of laboratory experiments, where limited space may inflate heterospecific interaction rates and they argue that more field studies are needed to understand the relevance and significant of such interactions in nature. Here we show that heterospecific courtship occurs at high rates among four species of sympatric *Habronattus* jumping spiders in the field and that these interactions can lead to female aggression and even predation. This high cost of misdirected courtship may help explain the evolution of colorful and complex multimodal communication of male *Habronattus* jumping spiders.

## Supporting information

S1 Fig Variation in coloration in male *Habronattus hirsutus*. (a) Male *H*. *hirsutus* with a completely black face (a) and a male *H*. *hirsutus* with a bright red facial patch (b). Over 95% of the males observed in our focal population at the Rio Salado Habitat Restoration Area (RSHRA) population were the black-faced form but occasionally males with bright red facial patches were found. All of the focal *H*. *hirsutus* males used for our study were the black-faced form.
(TIF)

## References

[1] Andersson M. Sexual selection. Princeton, NJ: Princeton University Press; 1994 443 p.

[2] Hoback WW, Wagner WE. The energetic cost of calling in the variable field cricket, *Gryllus lineaticeps*. Physiological Entomology. 1997;22(3):286–90.

[3] Wells KD, Taigen TL. Calling energetics of a neotropical treefrog, *Hyla microcephala*. Behavioral Ecology and Sociobiology. 1989;25(1):13–22.

[4] Cady AB, Delaney KJ, Uetz GW. Contrasting energetic costs of courtship signaling in two wolf spiders having divergent courtship behaviors. Journal of Arachnology. 2011;39(1):161–5.

[5] Cordts R, Partridge L. Courtship reduces longevity of male *Drosophila melanogaster*. Animal Behaviour. 1996;52:269–78.

[6] South SH, Steiner D, Arnqvist G. Male mating costs in a polygynous mosquito with ornaments expressed in both sexes. Proceedings of the Royal Society Biological Sciences Series B. 2009;276(1673):3671–8. 10.1098/rspb.2009.0991 PMC2817312 19640881

[7] Woods WA, Hendrickson H, Mason J, Lewis SM. Energy and predation costs of firefly courtship signals. American Naturalist. 2007;170(5):702–8. 10.1086/521964 17926292

[8] Clark DL, Zeeff CK, Karson A, Roberts JA, Uetz GW. Risky Courtship: Background contrast, ornamentation, and display behavior of wolf spiders affect visual detection by toad predators. Ethology. 2016;122(5):364–75.

[9] Groening J, Hochkirch A. Reproductive interference between animal species. Quarterly Review of Biology. 2008;83(3):257–82. 18792662 10.1086/590510

[10] Gwynne DT, Rentz DCF. Beetles on the bottle—male buprestids mistake stubbies for females (Coleoptera). Journal of the Australian Entomological Society. 1983;22:79–80.

[11] Andrews RH, Petney TN, Bull CM. Reproductive interference between three parapatric species of reptile tick. Oecologia. 1982;52(2):281–6. 28310522 10.1007/BF00363851

[12] Costa-Schmidt LE, Machado G. Reproductive interference between two sibling species of gift-giving spiders. Animal Behaviour. 2012;84(5):1201–11.

[13] Fea MP, Stanley MC, Holwell GI. Fatal attraction: sexually cannibalistic invaders attract naive native mantids. Biology Letters. 2013;9(6). 10.1098/rsbl.2013.0746 PMC3871360 24284560

[14] Hettyey A, Vagi B, Kovacs T, Ujszegi J, Katona P, Szederkenyi M, et al Reproductive interference between Rana dalmatina and Rana temporaria affects reproductive success in natural populations. Oecologia. 2014;176(2):457–64. 10.1007/s00442-014-3046-z 25138258

[15] Kyogoku D, Sota T. Exaggerated male genitalia intensify interspecific reproductive interference by damaging heterospecific female genitalia. J Evol Biol. 2015;28(6):1283–9. 10.1111/jeb.12646 25882439

[16] Quinones-Lebron SG, Kralj-Fiser S, Gregoric M, Lokovsek T, Candek K, Haddad CR, et al Potential costs of heterospecific sexual interactions in golden orbweb spiders (*Nephila spp*.). Scientific Reports. 2016;6. 10.1038/srep36908 PMC5109271 27845369

[17] Jackson RR, Pollard SD. Jumping spider mating strategies: sex among cannibals in and out of webs In: Choe JC, Crespi BJ, editors. Mating systems in insects and arachnids. Cambridge, UK: Cambridge University Press; 1997 p. 340–51.

[18] Maddison W, Hedin M. Phylogeny of *Habronattus* jumping spiders (Araneae: Salticidae), with consideration of genital and courtship evolution. Systematic Entomology. 2003;28(1):1–21.

[19] Maddison W, McMahon M. Divergence and reticulation among montane populations of a jumping spider (*Habronattus pugillis* Griswold). Systematic Biology. 2000;49(3):400–21. 12116419 10.1080/10635159950127312

[20] Taylor LA. Color and communication in *Habronattus* jumping spiders: tests of sexual and ecological selection. Tempe: Arizona State University; 2012.

[21] Richman DB. Comparative studies on the mating behavior and morphology of some species of Pellenes (Araneae, Salticidae) [M.S. thesis]. Tucson, AZ: University of Arizona; 1973.

[22] Cutler B. Courtship behavior in *Habronattus captiosus* (Araneae, Salticidae). Great Lakes Entomologist. 1988;21(3):129–31.

[23] Maddison WP, Stratton GE. Sound production and associated morphology in male jumping spiders of the *Habronattus agilis* species group (Araneae, Salticidae). Journal of Arachnology. 1988;16(2):199–211.

[24] Elias DO, Hebets EA, Hoy RR, Mason AC. Seismic signals are crucial for male mating success in a visual specialist jumping spider (Araneae: Salticidae). Animal Behaviour. 2005;69:931–8.

[25] Elias DO, Land BR, Mason AC, Hoy RR. Measuring and quantifying dynamic visual signals in jumping spiders. Journal of Comparative Physiology A Sensory Neural and Behavioral Physiology. 2006;192(8):785–97. 10.1007/s00359-006-0116-7 16544164

[26] Taylor LA, Clark DL, McGraw KJ. Condition dependence of male display coloration in a jumping spider (*Habronattus pyrrithrix*). Behavioral Ecology and Sociobiology. 2011;65(5):1133–46.

[27] Taylor LA, Clark DL, McGraw KJ. From spiderling to senescence: ontogeny of color in the jumping spider, *Habronattus pyrrithrix*. Journal of Arachnology. 2014;42(3):268–76.

[28] Bulbert MW, O'Hanlon JC, Zappettini S, Zhang S, Li D. Sexually selected UV signals in the tropical ornate jumping spider, *Cosmophasis umbratica* may incur costs from predation. Ecology and Evolution. 2015;5(4):914–20. 10.1002/ece3.1419 25750717 PMC4338973

[29] Griswold CE. A revision of the jumping spider genus *Habronattus* F.O.P. Cambridge (Araneae: Salticidae), with phenetic and cladistic analyses. University of California Publications in Entomology. 1987;107:1–344.

[30] Endler JA. Variation in the appearance of guppy color patterns to guppies and their predators under different visual conditions. Vision Research. 1991;31(3):587–608. 1843763 10.1016/0042-6989(91)90109-i

[31] Endler JA. Signals, signal conditions, and the direction of evolution. American Naturalist. 1992;139:S125–S53.

[32] Endler JA. The color of light in forests and its implications. Ecological Monographs. 1993;63(1):1–27.

[33] Endler JA, Thery M. Interacting effects of lek placement, display behavior, ambient light, and color patterns in three neotropical forest-dwelling birds. American Naturalist. 1996;148(3):421–52.

[34] Elias DO, Mason AC, Hoy RR. The effect of substrate on the efficacy of seismic courtship signal transmission in the jumping spider *Habronattus dossenus* (Araneae: Salticidae). Journal of Experimental Biology. 2004;207(23):4105–10. 15498956 10.1242/jeb.01261

[35] Ptacek MB. The role of mating preferences in shaping interspecific divergence in mating signals in vertebrates. Behav Processes. 2000;51(1–3):111–34. 11074316 10.1016/s0376-6357(00)00123-6

[36] Losos JB. An experimental demonstration of the species-recognition role of anolis dewlap color. Copeia. 1985;(4):905–10.

[37] Warburton K, Lees N. Species discrimination in guppies: Learned responses to visual cues. Animal Behaviour. 1996;52:371–8.

[38] World Spider Catalog. World Spider Catalog. Natural History Museum Bern, online at http://wsc.nmbe.ch; 2016 [cited 2016 November 17th].

[39] Zurek DB, Cronin TW, Taylor LA, Byrne K, Sullivan MLG, Morehouse NI. Spectral filtering enables trichromatic vision in colorful jumping spiders. Current Biology. 2015;25(10):R403–R4. 10.1016/j.cub.2015.03.033 25989075

[40] Richman DB. Epigamic display in jumping spiders (Araneae, Salticidae) and its use in systematics. Journal of Arachnology. 1982;10(1):47–67.

[41] Elias DO, Maddison WP, Peckmezian C, Girard MB, Mason AC. Orchestrating the score: complex multimodal courtship in the *Habronattus coecatus* group of *Habronattus* jumping spiders (Araneae: Salticidae). Biological Journal of the Linnean Society. 2012;105(3):522–47.

[42] Masta SE, Maddison WP. Sexual selection driving diversification in jumping spiders. Proceedings of the National Academy of Sciences of the United States of America. 2002;99(7):4442–7. 10.1073/pnas.072493099 11930004 PMC123667

[43] Taylor LA, McGraw K. Male ornamental coloration improves courtship success in a jumping spider, but only in the sun. Behavioral Ecology 2013;24(4):955–67.

[44] Rio Salado habitat restoration area: history and restoration. http://phoenix.gov/recreation/rec/parks/preserves/locations/riosalado/history/index.html. Accessed 13 August 2011. [Internet]. 2011. http://phoenix.gov/recreation/rec/parks/preserves/locations/riosalado/history/index.html.

[45] Jackson RR. The behavior of communicating jumping spiders In: Witt PN, Rovner JS, editors. Spider communication: mechanisms and ecological significance. Princeton, NJ: Princeton University Press; 1982.

[46] Foelix RN. Biology of Spiders. New York, NY: Oxford University Press; 1996.

[47] Groening J, Luecke N, Finger A, Hochkirch A. Reproductive interference in two ground-hopper species: testing hypotheses of coexistence in the field. Oikos. 2007;116(9):1449–60.

[48] Blackburn GS, Maddison WP. Insights to the mating strategies of *Habronattus americanus* jumping spiders from natural behaviour and staged interactions in the wild. Behaviour. 2015;152(9):1168–87.

[49] Arnqvist G, Rowe L. Sexual conflict. Princeton, NJ, USA: Princeton University Press; 2005.

[50] Mappes J, Alatalo RV, Kotiaho J, Parri S. Viability costs of condition-dependent sexual male display in a drumming wolf spider. Proceedings of the Royal Society Biological Sciences Series B. 1996;263(1371):785–9.

[51] Kotiaho JS. Testing the assumptions of conditional handicap theory: costs and condition dependence of a sexually selected trait. Behavioral Ecology and Sociobiology. 2000;48(3):188–94.

[52] Hoefler CD. The costs of male courtship and potential benefits of male choice for large mates in *Phidippus clarus* (Araneae, Salticidae). Journal of Arachnology. 2008;36(1):210–2.

[53] Hoefler CD, Persons MH, Rypstra AL. Evolutionarily costly courtship displays in a wolf spider: a test of viability indicator theory. Behav Ecol. 2008;19(5):974–9.

[54] Su KFY, Li DQ. Female-biased predation risk and its differential effect on the male and female courtship behaviour of jumping spiders. Animal Behaviour. 2006;71:531–7.

[55] Arnqvist G. The evolution of water strider mating systems: causes and consequences of sexual conflicts In: Choe JC, Crespi BJ, editors. Mating systems in insects and arachnids. Cambridge, UK: Cambridge University Press; 1997 p. 146–63.

[56] Watson PJ, Arnqvist G, Stallmann RR. Sexual conflict and the energetic costs of mating and mate choice in water striders. American Naturalist. 1998;151(1):46–58. 10.1086/286101 18811423

[57] Lim MLM, Land MF, Li DQ. Sex-specific UV and fluorescence signals in jumping spiders. Science. 2007;315:481-. 10.1126/science.1134254 17255504

[58] Lim MLM, Li JJ, Li D. Effect of UV-reflecting markings on female mate-choice decisions in *Cosmophasis umbratica*, a jumping spider from Singapore. Behavioral Ecology. 2008;19:61–6.

[59] Li JJ, Zhang ZT, Liu FX, Liu QQ, Gan WJ, Chen J, et al UVB-based mate-choice cues used by females of the jumping spider *Phintella vittata*. Current Biology. 2008;18(9):699–703. 10.1016/j.cub.2008.04.020 18450445

[60] Cross FR, Jackson RR, Pollard SD. How blood-derived odor influences mate-choice decisions by a mosquito-eating predator. Proceedings of the National Academy of Sciences of the United States of America. 2009;106(46):19416–9. 10.1073/pnas.0904125106 19887641 PMC2780784

[61] Nelson XJ, Warui CM, Jackson RR. Widespread reliance on olfactory sex and species identification by lyssomanine and spartaeine jumping spiders. Biological Journal of the Linnean Society. 2012;107(3):664–77.

[62] Hochkirch A, Groening J, Buecker A. Sympatry with the devil: reproductive interference could hamper species coexistence. Journal of Animal Ecology. 2007;76(4):633–42. 10.1111/j.1365-2656.2007.01241.x 17584368

