# Supplementary figures and images for: Frequent misdirected courtship in a natural community of colorful Habronattus jumping spiders
Source: PLoS One. 2017 Apr 5;12(4):e0173156. doi: 10.1371/journal.pone.0173156 (PMC5381776; doi:10.1371/journal.pone.0173156)

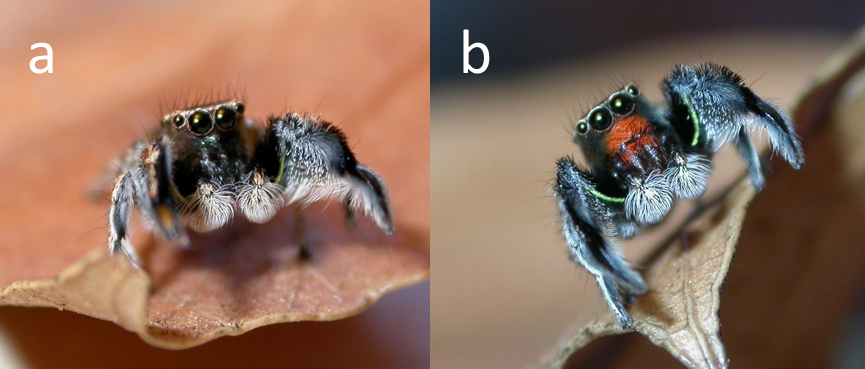

Supplement: S1 Fig — (a) Male H. hirsutus with a completely black face (a) and a male H. hirsutus with a bright red facial patch (b). Over 95% of the males observed in our focal population at the Rio Salado Habitat Restoration Area (RSHRA) population were the black-faced form but occasionally males with bright red facial patches were found. All of the focal H. hirsutus males used for our study were the black-faced form. (TIF) [file pone.0173156.s001.tif]
